# Genetic inbreeding depression load for fertility traits in Pura Raza Española mares

**DOI:** 10.1093/jas/skab316

**Published:** 2021-10-29

**Authors:** Davinia I Perdomo-González, Antonio Molina, María J Sánchez-Guerrero, Ester Bartolomé, Luis Varona, Mercedes Valera

**Affiliations:** 1 Departamento de Agronomía, ETSIA, Universidad de Sevilla, Sevilla, Spain; 2 Departamento de Genética, Universidad de Córdoba, Córdoba, Spain; 3 Departamento de Biología Molecular e Ingeniería Bioquímica, Universidad Pablo de Olavide, Sevilla, Spain; 4 Departamento de Anatomía Embriología y Genética Animal, Instituto Agroalimentario de Aragón (IA2), Universidad de Zaragoza, Zaragoza, Spain

**Keywords:** fertility, genetic parameters, horses, inbreeding depression, inbreeding load heterogeneity, reproductive efficiency

## Abstract

Fertility is a key factor in the economic success of horse farms. However, it has received little attention due to the difficulty of measuring fertility objectively. Since its studbook creation (1912), the Pura Raza Española (PRE) breed has been a closed population and become high in-bred resulting in inbreeding depression (poor phenotypic values). Nevertheless, heterogeneous effects of inbreeding depression have been detected among founders and nonfounders. The aims of this study were (1) to analyze the genetic parameters for reproductive traits in mares of the PRE horse breed and (2) to estimate, for the first time, the inbreeding depression load associated with common ancestors of the breed. A total of 22,799 mares were analyzed. Heritability estimates ranged from 0.05 (interval between first and second foaling) to 0.16 (age at first foaling), whereas inbreeding depression load ratios ranged from 0.06 (parturition efficiency at 6th foaling) to 0.17 (age at first foaling), for a partial inbreeding coefficient of 10%. Although heritability is related to the variability expressed in the population, inbreeding depression load ratios measure the potential variability, whether expressed in the population or not. Most correlations between additive and inbreeding depression load genetic values were significant (*P* < 0.001) and of low to moderate magnitude. Our results confirm that individual inbreeding depression loads allow us to select horses that have a genetic value resistant to the deleterious effects of inbreeding.

## Introduction

Fertility is the capacity to conceive or to induce conception in order to produce offspring and contribute to the next generation. Not only is fertility crucial for a populations’ survival, but it has also been related to the economic success of the farm, regardless of the species or breed (Wolc et al., 2009). Over the last few decades, fertility has been studied as a breeding goal in domestic species for beef and milk production (Meyer et al., 1990; Phocas et al., 1998; Liu et al., 2008; Robinson, 2008; VanRaden et al., 2014; Dezetter et al., 2015). However, equines have historically been selected for conformation or performance in equestrian sports without paying much attention to fertility (Sairanen et al., 2009; Wolc et al., 2009; Stock et al., 2015). In fact, what has determined the animal chosen for mating has been a combination of the horse’s performance, pedigree, and certain morphological traits. For this reason, there is little literature available on fertility traits and their inclusion as selection criteria in equine breeding programs, although some authors have reported a genetic influence on such traits (Taveira and Dias, 2007; Sairanen et al., 2009; Wolc et al., 2009; Sabeva and Apostolov, 2011; Mucha et al., 2012; Gómez et al., 2020; Mantovani et al., 2020).

Heritabilities for fertility traits have shown low values in several species (Cammack et al., 2009) including horses (Taveira and Dias, 2007; Sairanen et al., 2009; Gómez et al., 2020). Fertility is affected by a wide variety of management, environmental, and genetic issues, which make it difficult to identify factors directly linked to the animal’s genetic merit. The main factors studied affecting fertility are month of mating, age of stallion, age of mare, status of mare, and mating type (Müller-Unterberg et al., 2017), but the most important one is inbreeding (F) (Hevia et al., 1994; Morris and Allen, 2002; Sairanen et al., 2009; Müller-Unterberg et al., 2017).

The most outstanding individuals are normally the most frequently selected as parents for breeding. Consequently, there is an uneven contribution of genes to the next generation. Thus, breeders face increased homozygosity (inbreeding) in their populations which has been associated with a reduction in animal fitness and of performance, especially of reproductive capacity (Leroy, 2014). This reduction in animal fitness is due to reduced genetic heterozsity and the exposure to recessive deleterious mutations (Charlesworth and Willis, 2009). This is referred to as inbreeding depression. Frequently, the effects of inbreeding depression have been analyzed assuming a linear relationship with phenotypic values in the absence of epistasis. However, this approach does not take into account important factors such as the uneven distribution of the recessive genetic load transmitted by different founders, the different founders’ selection pressures, or the large number of loci involved (Latter and Robertson, 1962; Latter, 1998). In the last few decades, the variability of inbreeding depression has been confirmed in different breeds, mainly for growth or milk traits (Miglior et al. 1994; Carolino and Gama, 2008; Casellas et al., 2009; Varona et al., 2019). To date, however, only a few articles have analyzed inbreeding depression in horses, and these have focused on racing performance (Todd et al., 2018) and morphology (Gómez et al., 2009; Poyato-Bonilla et al., 2020). Although these studies have revealed deleterious effects due to inbreeding depression, inbreeding actually shows an extensive range of negative, neutral, and positive effects in livestock. This is known as inbreeding depression load (Varona et al., 2019) and is calculated by assessing the hidden inbreeding loads associated with founders and the Mendelian sampling of non-founders.

Pura Raza Española horse (PRE) is one of the oldest European horse breeds and is present today in over 60 countries, with up to 260,000 active horses (Asociación Nacional de Criadores de Caballos de Pura Raza Española, ANCCE). One of its most highly prized characteristics is its versatility for dressage and sports, with a harmonious shape, good psychological balance, and a willingness to work. Despite the large population, genealogical analysis has shown that the breed was derived from a few founder individuals and was developed through selection of certain desired traits often mating close relatives. As a result, inbreeding levels are extremely high (Valera et al., 2005; Poyato-Bonilla et al., 2018). In fact, the average PRE inbreeding is 7.25%, with over 46% of the current population having an inbreeding coefficient equal or higher than 6.25% (Perdomo-González et al., 2020).

The aims of the present study were, firstly, to estimate genetic parameters and the level of inbreeding depression for six reproductive traits and three reproductive efficiency parameters in PRE mares and, secondly, to perform a genetic analysis of the inbreeding depression load considering the partial inbreeding coefficients (Fij) of the common ancestors.

## Materials and Methods

### Data set

The pedigree data were obtained from the ANCCE studbook, for 344,707 animals (168,296 males and 176,411 females) born from the early 1900s to 2019, among which 103,675 animals were used as breeders (24,468 stallions and 79,207 mares). As early as the 1970s, paternity controls were being carried out in PRE by blood grouping or serum polymorphisms, showing a filiation success of over 70% (de Andres Cara and Kaminsky, 1984; Kaminski and De Andres, 1986). For this reason, from the mares with descendants, we selected those mares born after 1970 who had their first foal on a farm whose main activity was the production of foals (over 12 foals per year). Also, to exclude those mares dedicated to leisure or sport, we selected mares who had their first foal between 4 and 7 yr old, an interval between first and second foaling equal or below 5 yr, an average interval between foaling over 9 mo and an interval between the last and penultimate foaling below 5 yr, thus increasing the consistency of the data. The final database contained reproductive information from 22,799 mares born from 1970 to 2015. From the selected mares, a pedigree file with all the available generations was generated. This contained 39,003 horses (6913 males and 32,090 females) as the evaluated population.

In this study, we analyzed six reproductive traits and two different approaches for reproductive efficiency. The reproductive traits were total number of foalings (FN), age at first foaling (AFF), average interval between first and second foaling (I12), average interval between foaling (AIF), age at last foaling (ALF), and productive life (PL, defined as the years the animal had been actively breeding). PL and ALF were only recorded for mares that had finished their reproductive period; mares that were at least 25 yr old or at least 20 yr old without any parturition in the last 5 yr. All the traits were measured in months, except FN. The two new approaches for reproductive efficiency were global reproductive efficiency (GE) and parturition efficiency (PE). GE is the number of total foalings relative to the optimal number of foalings the mare could have during her entire life (ONTF). PE is the number of foalings the mare has had in a specific moment (FNm) relative to the number of optimal foalings at that mare’s age (ONFm). PE allows us to analyze the mare’s reproductive efficiency for each foaling moment. To compare reproductive efficiency at two moments in the reproductive life of mares, we analyze the reproductive efficiency when the mare has had its third foaling (PE3) and sixth foaling (PE6), respectively. ONTF was calculated adding one (for the first foaling) to the difference between the age at last foaling (in years) and the age at the first foaling (assuming as optimum a foal every year from the first foaling). ONFm was calculated adding one (for the first foaling) to the difference between the age (in years) at this foaling and the age at the first foaling (assuming as optimum a foal every year from the first foaling). Finally, GE was calculated as ONTF minus FN, then it is divided by ONTF, and the result is rested to 1, to finally multiplied by 100.In the same way, PE was calculated as ONFm minus FNm, then it is divided by ONFm, and the result is rested to 1, to finally multiplied by 100.

### Inbreeding estimation

Wright’s coefficient of inbreeding (*F*, also known as classical inbreeding) is defined as the probability that an individual possesses two identity-by-descendant (IBD) alleles at a randomly chosen locus. The recent inbreeding coefficient at the 3rd generation (F3) was computed following the methods described by Meuwissen and Luo (1992) using the Endog v4.8 program (Gutierrez and Goyache, 2005). The ancestral inbreeding coefficient of Kalinowski (Fk) (Kalinowski et al., 2000) is defined as the probability that any allele in an individual is currently autozygous and has been autozygous (IBD) at least once in preceding generations was calculated with GRain 2.2 software (Doekes et al., 2020). Finally, partial inbreeding coefficients (Fij), the combined probability that an individual i is autozygous for an allele and that this allele was from ancestor j (Lacy et al., 1996; Rodrigáñez et al., 1998), were calculated following the methodology of Casellas (2018) for all the individuals in the evaluated population. This approach partitioned the inbreeding into the sources of the coancestry between the parents of each individual, considering the founders of the population and the Mendelian sampling of the non-founders (Caballero and Toro, 2000; García-Cortés et al., 2010). Thus, 4,171,147 Fij coefficients were derived from 3581 common ancestors (founders and non-founder belonging both to the paternal and maternal lines).

### Statistical analyses

The data for each reproductive trait (**y**) were analyzed using a linear model proposed by Casellas (2018) and reformulated by Varona et al. (2019). In this model, the standard breeding value and the inbreeding depression load generated by the animals’ ancestors are used as random genetic effects to explain the phenotypic values and reflect the additive nature of the individual inbreeding depression load (**i**).

The final model can be represented as


Y=fc+ Xb + Zu + Ki + e

This model includes a vector of total inbreeding of analyzed mares (**f**), a covariate with the total inbreeding depression (**c**), systematic effects (**b**), infinitesimal additive genetic contributions (**u**), individual inbreeding depression load effects (**i**), and residual terms (**e**). The prior distribution of the additive genetic, inbreeding depression load, and residual effects was


$$\left(\begin{array}{c} u\\ i \end{array}\right)\;\sim N\left(\begin{array}{c} 0\\ 0 \end{array},\;G\;⊗\;A\right)ande\;N(0,I\sigma_{e}^{2})$$

where $G\;=\;\left(\begin{array}{cc} \sigma_{u}^{2} & \sigma_{ui}\\ \sigma_{ui} & \sigma_{i}^{2}\;\;\; \end{array}\right)$; **A** is the numerator of relationship matrix; $\sigma_{u}^{2}$, $\sigma_{i}^{2}$, and $\sigma_{e}^{2}$ are the associated variance components; and $\sigma_{ui}$ is the covariance between the additive genetic and the inbreeding depression load effects, respectively. **X** and **Z** are incidence matrices of systematic effects and additive genetic contributions, respectively, and K=T(I−P). **T** is a lower triangular matrix in which each non-zero element is a Fij which links the phenotype of an inbred individual to each ancestor causing inbreeding and, for computational reasons, each Fij was multiplied by 10 to obtain the inbreeding depression load variance for a Fij of 10%. **P** is a projection matrix with a 0 diagonal and 0.5 in the elements that link an individual to its sire and dam, and **I** is the identity matrix. More specifically, **b** includes the coat color of the mare (6 levels), geographic stud zone (3 levels), and average owner stud size in the decade of the mare’s first foaling (15 levels except for PL and ALF, with 12 levels). Additionally, as quadratic covariates, ALF was included in the FN, AIF, PL, and GE models, and length of life in PE3 and PE6 models. Length of life was her age at death if she had already died. Otherwise, it was her age at her last foaling if she was 21 or older and it was her age at her last foaling plus 30 mo if she was less than 21 yr (to avoid overestimation for mares how had not finished her productive life). The MCMC model was implemented using ad hoc software written in FORTRAN90 (Varona et al., 2019) and one single chain of 5,500,000 samples and a burning period of 500,000 were used for each trait. This analysis will also allow us to calculate the inbreeding depression load ratios, which should be interpreted as the proportion of the variation of each phenotypic unit value in the population that is attributable to the variation in the inbreeding depression effects in a theoretical population where each of the individuals has a partial inbreeding coefficient generated by a single, specific ancestor (Varona et al., 2019; Poyato-Bonilla et al., 2020; Martinez-Castillero et al., 2021). Simple linear regression coefficients between the inbreeding coefficients (F3, F, and Fk) and the phenotypic values of analyzed traits were performed to assess the effect of inbreeding depression. Pearson’s correlations between the inbreeding depression load genetic values (IL) and between the breeding values (BV) and the IL were performed as estimators of genetic correlations between traits (Calo et al., 1973). All statistical analyses were made using the R software (R Development Core Team, 2020).

## Results

The descriptive statistics for the six reproductive traits and three reproductive efficiency parameters analyzed in PRE mares can be seen in Table 1. The reproductive trait means were 57.57 (AFF), 17.49 (I12), 18.80 (AIF), 5.30 (FN), 169.70 (ALF), and 113.40 (PL). FN was the trait with the highest coefficient of variation (69.52) and AFF the lowest (17.43). The means for reproductive efficiency parameters were 45.4 (GE), 77.50 (PE3), and 75.39 (PE6). GE had the highest coefficient of variation and PE6 the lowest (39.26 and 26.18, respectively).

**Table 1.** Descriptive statistics for six direct reproductive traits and three reproductive efficiency parameters in Pura Raza Española mares

	Number	Mean (SE)	CV(%)	Minimum	Maximum
AFF	22799	57.57 (0.07)	17.43	45	84
I12	19441	17.49 (0.06)	50.78	10	60
AIF	19441	18.80 (0.05)	36.96	9	110
FN	22799	5.30 (0.02)	69.52	1	22
ALF	9241	169.70 (0.60)	34.13	55	420
PL	9241	113.40 (0.61)	51.62	10	361
GE	22799	45.49 (0.12)	39.26	5	106
PE3	16439	77.50 (23.98)	30.95	12	150
PE6	9268	75.39 (0.21)	26.18	21	150

Age at first foaling in months (AFF), average interval between first and second foaling in months (I12), average interval between foaling in months (AIF), total number of foalings (FN), age at last foaling in months (ALF), productive life in months (PL), global efficiency in % (GE): parturition efficiency in % (PE) at 3rd and 6th foalings (PE3 and PE6, respectively).

**Figure 1.**
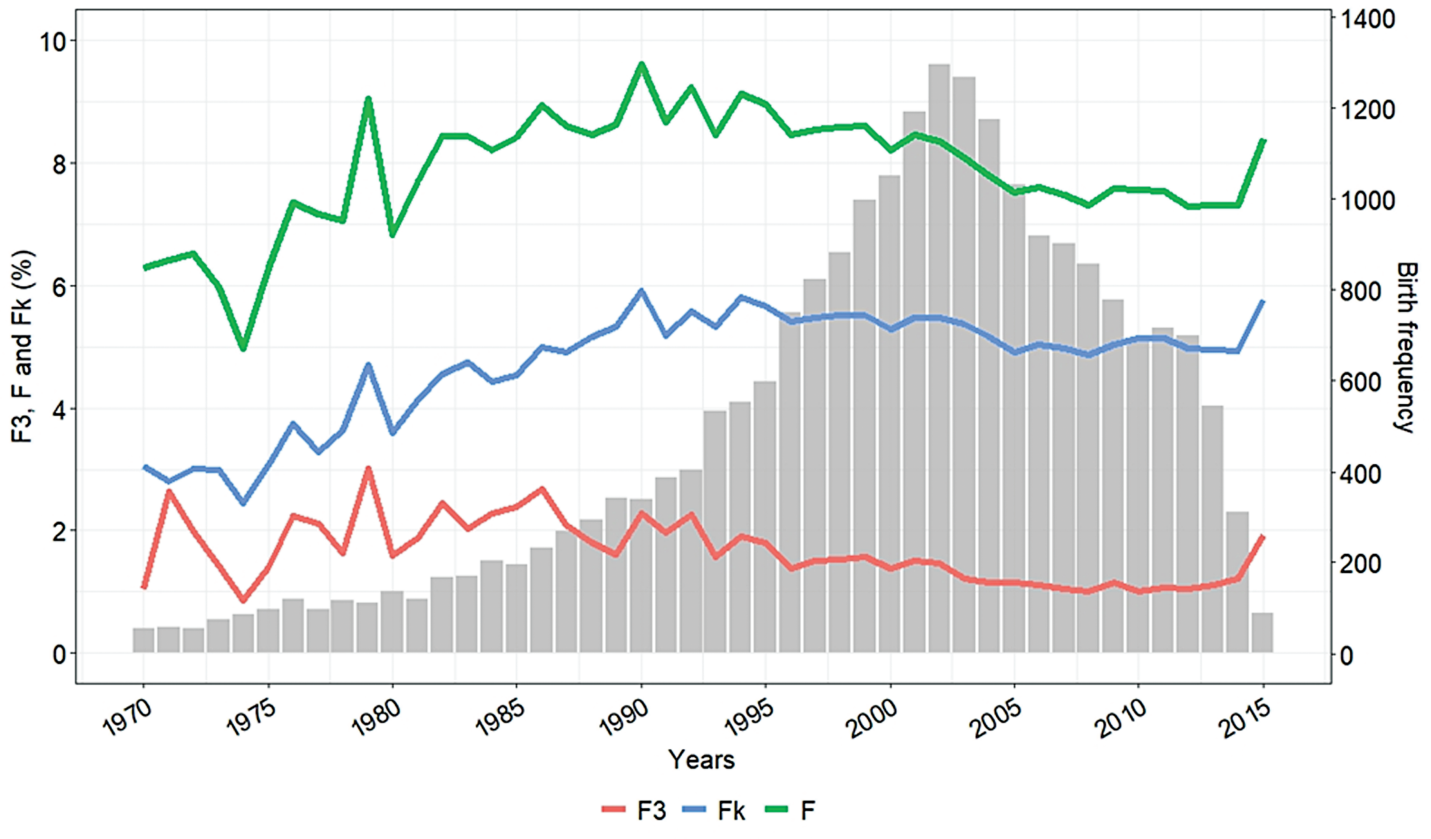
Annual levels of average recent inbreeding at third generation (F3%), ancestral Kalinowski inbreeding (Fk%), Wright’s coefficient of inbreeding (F%) of Pura Raza Española mares analyzed (22,799 mares), and birth frequency.

The change of average inbreeding coefficients over time for the analyzed mares is shown in Figure 1. Average F3, Fk, and *F* values showed similar patterns, increasing from 1970 to 1990 and then decreasing to remain relatively constant in the recent years. *F* values were the highest, with a maximum average value of 9.1% in 1990, decreasing to around 7% in recent years. Although F3 remained the lowest between 1% and 3%, Fk showed intermediate values closer to *F* than F3, with values around 5% over the last 20 yr. The number of mares used as breeders increased dramatically up until 2002, when the number began to decline sharply.

The simple linear regression coefficients between those three inbreeding coefficients (F3, Fk, and *F*) and reproductive traits and efficiency parameters were mostly significant (Table 2). The regression coefficient was positive for AFF, I12, and AIF where the higher the value of the trait, the lower the mare’s reproductive aptitude. For the other traits, the negative regression coefficient corresponds to poorer breeding performance. The breeders’ objective is to lower breeding intervals as much biologically as possible. Most regression coefficients were significant (*P* < 0.05). For AFF, I12, and AIF, the highest coefficients were found with Fk (13.65, 10.08, and 7.34, respectively), whereas for the other traits, coefficients with Fk were the lowest ranging from −235.61 (PL) to −9.62 (FN). All regression coefficients with *F* were also significant, the positive coefficients ranged from 4.33 (AIF) to 6.63 (AFF), and the negative ones ranged between −91.40 (PL) and −1.70 (FN). Meanwhile, the F3 coefficient showed a lower degree of significance in the regressions, whereas the regressions with the variables AFF, ALF, PL, and PE3 were not statistically significant. However, the regression sign was the same as for the other inbreeding coefficients except for FN.

**Table 2.** Simple regression coefficients between three inbreeding coefficients and the reproductive traits and reproductive efficiency parameter in Pura Raza Española inbred mares

	F3	Fk	F
AFF	0.44	13.65***	6.63***
I12	5.14**	10.08***	5.51***
AIF	3.31*	7.34***	4.33***
FN	2.13***	−9.62***	−1.70***
ALF	−4.15	−226.91***	−86.02***
PL	−3.56	−235.61***	−91.40***
GE	−9.30**	−52.27***	−23.43***
PE3	−6.49	−35.85***	−14.35***
PE6	−10.44*	−34.33***	−17.54***

Age at first foaling in months (AFF), average interval between first and second foaling in months (I12), average interval between foaling in months (AIF), total number of foalings (FN), age at last foaling in months (ALF), productive life in months (PL), global efficiency in % (GE), parturition efficiency in % (PE) at 3rd and 6th foalings (PE3 and PE6, respectively). Inbreeding at the 3rd generation (F3), Kalinowski’s inbreeding coefficient (Fk), Wright’s inbreeding coefficient (F). ***P < 0.001; **P < 0.01; *P < 0.05.

Table 3 shows the heritability estimates, inbreeding depression load ratios and the variances attributed to the direct additive genetic effect, inbreeding depression load (corresponding to an inbreeding value of 10%), and the residual effect of the analyzed traits and efficiency parameters. Analogously to heritability, inbreeding depression load ratios were calculated as the relative magnitude of the inbreeding depression load variances. The heritability estimates were highest for AFF (0.16) and AIF (0.14), whereas the other estimates ranged from 0.05 (I12) to 0.10 (GE). Inbreeding depression load ratios, calculated for an inbreeding value of 10%, ranged from 0.06 (PE3) to 0.17 (AFF). For the most variables, the inbreeding depression load variance values were higher than the direct additive genetic variances, as a direct consequence of the arbitrary scaling of Fij by 10.

**Table 3.** Heritabilities, inbreeding load ratios, and additive, depression load, and residual variances in reproductive traits and efficiency parameters in the Pura Raza Española horse

			σ _u_			σi			σ _e_		
	*h* ^*2*^	*d* ^*2*^	Mean (SD)	Median	HPD_95%_	Mean (SD)	Median	HPD_95%_	Mean (SD)	Median	HPD_95%_
AFF	0.16	0.17	18.06 (1.33)	18.03	15.48, 20.68	20.24 (8.06)	19.61	5.31, 35.95	78.48 (1.25)	78.49	76.02, 80.94
I12	0.05	0.12	4.50 (0.86)	4.46	2.88, 6.24	10.92 (5.75)	10.09	1.71, 22.50	73.47 (1.06)	73.48	71.40, 75.54
AIF	0.14	0.13	6.68 (0.87)	6.66	5.01, 8.41	4.34 (3.07)	3.87	0.10, 10.24	40.82 (0.81)	40.83	39.24, 42.4
FN	0.08	0.10	0.21 (0.02)	0.21	0.17, 0.26	0.27 (0.15)	0.26	0.02, 0.53	2.24 (0.03)	2.24	2.18, 2.30
ALF	0.06	0.10	163.05 (35.11)	161.23	97.04, 233.32	285.44 (177.58)	255.61	26.43, 624.09	2389.25 (46.57)	2389.26	2298.29, 2480.85
PL	0.08	0.14	19.50 (3.38)	19.36	12.95, 26.15	31.62 (17.99)	29.10	2.26, 64.85	182.46 (3.90)	182.46	174.80, 190.09
GE	0.10	0.09	21.34 (2.23)	21.29	17.01, 25.76	19.67 (11.93)	18.53	0.64, 41.03	170.11 (2.42)	170.11	165.36, 174.84
PE3	0.06	0.06	36.27 (7.25)	35.90	22.42, 50.64	35.86 (27.32)	30.56	0.59, 89.19	513.00 (8.43)	513.06	496.35, 529.37
PE6	0.07	0.10	25.51 (6.54)	25.10	13.37, 38.62	42.25 (29.64)	37.03	1.85, 99.66	357.42 (7.80)	357.50	342.03, 372.54

*h* ^2^, heritabilities; *d*^2^, inbreeding depression load ratios; σu, direct additive genetic variances; σ _i_, inbreeding depression load variances; σ _e_, residual variances; SD, standard deviation; HPD_95%_, highest posterior density at 95%. Values correspond to a horse with an inbreeding value of 10%. Total number of foalings (FN), age at first foaling in months (AFF), average interval between first and second foaling in months (I12), average interval between foaling in months (AIF), age at last foaling in months (ALF), productive life in months (PL), global efficiency in % (GE), parturition efficiency in % (PE) at 3rd and 6th foalings (PE3 and PE6, respectively).

**Figure 2.**
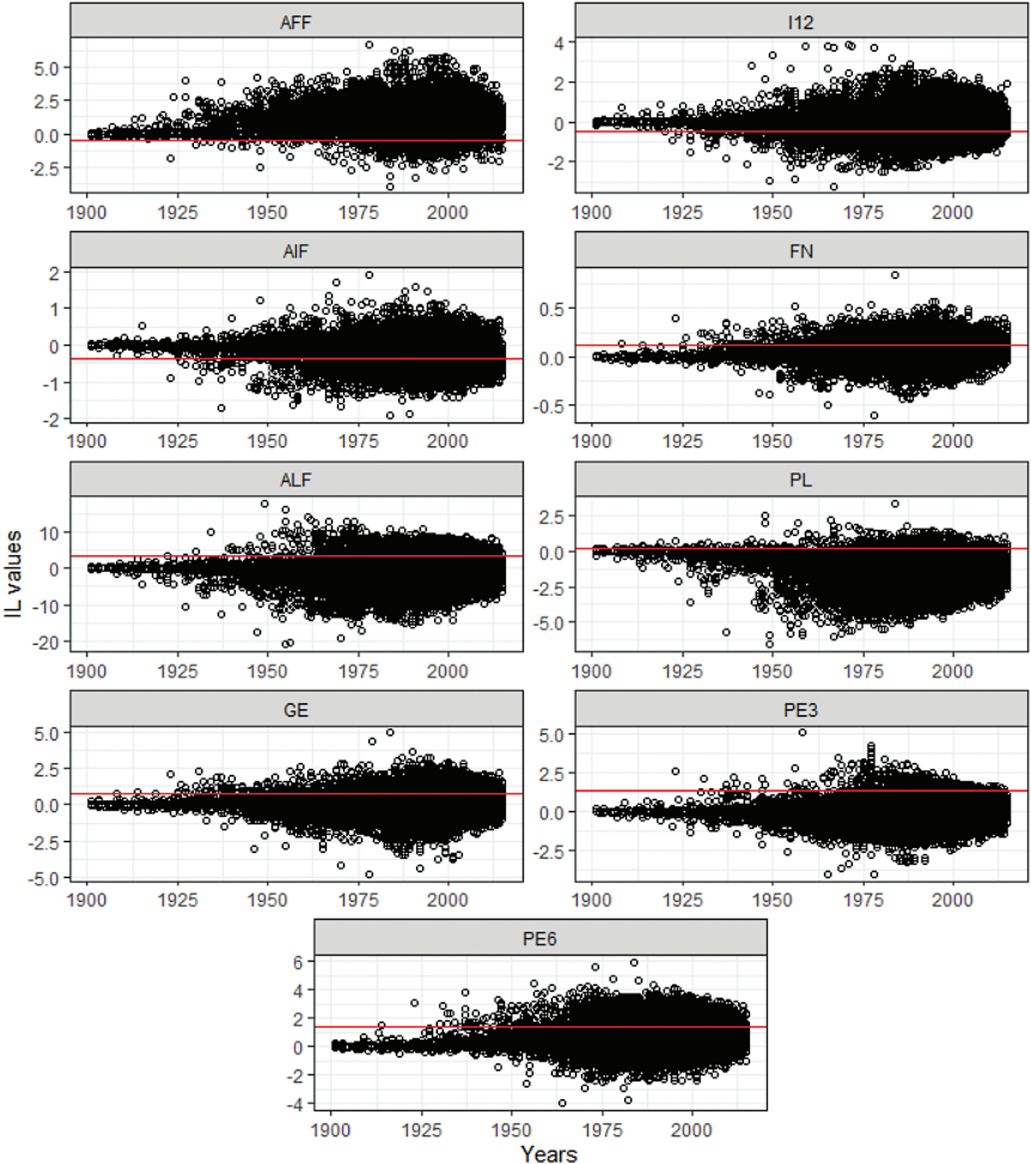
Inbreeding depression load variation and its average annual level for all analyzed traits in the Pura Raza Española horse population evaluated. Inbreeding load (IL) genetic value, age at first foaling in months (AFF), age at last foaling in months (ALF), average interval between first and second foaling in months (I12), average interval between foaling in months (AIF), total number of foalings (FN), productive life in months (PL), global efficiency in % (GE), parturition efficiency in % (PE) at 3rd and 6th foaling (PE3 and PE6, respectively), and slope with the inbreeding (red line).

Figure 2 shows the IL over time, corresponding to an *F* of 10%, across generations in the evaluated population. As can be observed, IL were variable and fluctuating around zero. The slope of the regression of the genetic values against the individuals own inbreeding level, which indicates the average effect of inbreeding, is also shown in Figure 2. The slope was positive for AFF, I12, and AIF (0.54, 0.47, and 0.36, respectively) and negative for FN, ALF, PL, GE, PE3, and PE6 (−0.11, −3.40, −0.22, −0.78, −1.34, and −1.42, respectively). The effect of inbreeding depression on a trait should be positive or negative depending on whether inbreeding load exceeds minus the value of the average slope, which implies that the individual inbreeding load compensates for the average effect of the inbreeding.

Finally, we estimated the Pearson’s correlations between BV and IL values (upper half of Table 4) and among IL values (lower half of Table 4). The diagonal shows the correlations between BV and IL for the same trait. Here, all values were significant and ranged from negative values, −0.17 (FN) and −0.11(PE1), to a highly positive values, 0.54 (ALF) and 0.27 (PE3), for reproductive traits and efficiency parameters, respectively. The correlations between BV and IL for different traits were mostly significant, positive, and close to zero. The highest positive correlations can be seen between IL_ALF and BV_FN (0.36), BV_GE (0.38), BV_PE3 (0.36) and BV_PE6 (0.44) and between IL_PL and BV_FN (0.43), BV_GE (0.34), BV_PE3 (0.38) and BV_PE6 (0.43), whereas the strongest negative correlations were between IL_AFF and BV_FN (−0.35) and BV_PE3 (−0.32) and between IL_ALF and BV_I12 (−0.31). At the same time, the Pearson’s correlations between IL values (lower part of Table 4) were significant and, in general, negative. The strongest positive correlations were between IL_FN and IL_GE (0.73) and IL_I12 and IL_AIF (0.65) and the strongest negative correlations were between IL_AIF and IL_FN (−0.71) and IL_PE6 (−0.68).

**Table 4.** Pearson’s correlations between estimated breeding values and inbreeding load genetic values (within and between traits) and Pearson’s correlations between inbreeding load genetic values in Pura Raza Española mares

	IL_AFF	IL_I12	IL_AIF	IL_FN	IL_ALF	IL_PL	IL_GE	IL_PE3	IL_PE6
BV_AFF	0.18*	0.01	−0.01	0.00	−0.09*	−0.16*	−0.02*	−0.08*	0.05*
BV_I12	0.19*	0.09*	−0.08*	0.07*	−0.31*	−0.27*	−0.01	−0.12*	0.06*
BV_AIF	0.21*	0.03*	−0.05*	0.08*	−0.27*	−0.23*	−0.01	−0.07*	0.14*
BV_FN	−0.35*	0.05*	0.18*	−0.17*	0.36*	0.43*	−0.04*	0.10*	−0.26*
BV_ALF	−0.16*	0.04*	−0.04*	0.02*	0.54*	0.30*	0.18*	0.21*	0.04*
BV_PL	−0.17*	0.02*	0.08*	−0.07*	0.06*	0.25*	0.07*	0.09*	−0.13*
BV_GE	−0.25*	0.09*	0.08*	−0.08*	0.38*	0.34*	0.10*	0.11*	−0.18*
BV_PE3	−0.32*	−0.02*	0.05*	−0.01	0.36*	0.38*	0.08*	0.27*	−0.10*
BV_PE6	−0.26*	0.03*	0.16*	−0.10*	0.44*	0.43*	−0.01	0.20*	−0.11*
IL_AFF		0.07*	−0.03*	−0.07*	−0.27*	−0.56*	−0.12*	−0.38*	0.07*
IL_I12			0.65*	−0.38*	0.00	0.16*	−0.39*	−0.51*	−0.51*
IL_AIF				−0.71*	0.03*	0.31*	−0.67*	−0.53*	−0.68*
IL_FN					−0.28*	−0.07*	0.73*	0.53*	0.63*
IL_ALF						0.39*	0.02*	0.24*	0.08*
IL_PL							0.15*	0.23*	−0.36*
IL_GE								0.59*	0.48*
IL_PE3									0.50*

Breeding value (BV), inbreeding load genetic value (IL), age at first foaling in months (AFF), average interval between first and second foaling in months (I12), average interval between foaling in months (AIF), total number of foalings (FN), age at last foaling in months (ALF), productive life in months (PL), global efficiency in % (GE), parturition efficiency in % (PE) at 3rd and 6th foalings (PE3 and PE6, respectively). *Significant correlations, *P* < 0.001. Standard error was ≤0.005 in all cases.

## Discussion

The PRE horse has a closed breeding system in which only animals belonging to the breed can act as breeding animals. This protocol has led to the degree of kinship between the reproducers, although very high, being generalized. The mating of related individuals is inevitable, leading to inbreeding in their offspring. Increased inbreeding (genomic homozygosity) can lower fitness in two genetically distinct ways: losing the advantage from alleles with heterozygous superiority (“overdominance”) and the unmasking of partially recessive detrimental mutations (Charlesworth and Willis, 2009). This phenomenon is historically known as inbreeding depression and has always been considered as a negative event because its usual consequence is to worse phenotypic values over a wide range of traits. However, over the last few decades, more research has been carried out into the genetic nature of this phenomenon and how unfavorable alleles are transmitted from the common ancestors. This has revealed a heterogeneity in inbreeding depression among founder and non-founder individuals for different traits in several species (Miglior et al., 1994; Carolino and Gama, 2008; Casellas et al., 2009; Todd et al., 2018; Varona et al., 2019; Poyato-Bonilla et al., 2020). The results show that inbreeding depression sometimes has neutral or positive impact to phenotypes (Varona et al., 2019; Poyato-Bonilla et al., 2020; Martinez-Castillero et al., 2021). This evidence leads us to conclude that inbreeding depression is not homogeneous among individuals with the same level of homozygosity but depends on the proportion of inbreeding by descendant (IBD) alleles received from each ancestor. Our study deals with the estimation of individual inbreeding depression load for reproductive traits and reproductive efficiency parameters in PRE mares, following the methodology proposed by Varona (2019).

The traits analyzed in this study have been selected because they are considered important for the evaluation of the reproductive performance of mares (Gómez et al., 2020). AFF and ALF designate, respectively, the beginning and the end of the reproductive life of individuals. AIF and I12 measure the interval between foaling events and constitute indirect parameters related to fertility in many species, including horses. FN and PL are directly related to the length of the animal’s reproductive life. On the other hand, GE, PE3, and PE6, applied for the first time in equines, are used to measure fertility efficiency for the final, initial, or intermediate parturition, respectively.

Our results regarding the mean values of reproductive traits are similar to those of Gómez et al. (2020), who analyzed the reproductive parameters of PRE mares and seven other horse breeds (Arab Horse, Purebred Menorca Horse, Spanish Sport Horse, Anglo-Arab Horse, Spanish Trotter Horse, Jaca Navarra, and Burguete). These authors reported slightly higher values for AFF (64.6), I12 (20.0), and AIF (20.9) and lower values for ALF (125.7) and PL (81.3) in PRE mares, highlighting the PRE mares’ precocity compared with other Spanish breeds, as they showed the lowest values for all the traits among all the breeds analyzed. The average number of foals reported by Sabeva and Apostolov (2011) in Arabian broodmares (6.4) was higher than that reported here, whereas productive life, defined as the number of years between the stud entrance date and leaving date, showed an average of 9.17 yr (~110 mo), higher than all the breeds studied by Gómez et al. (2020), but similar to our results for PRE mares. These results support the idea that PRE breeders usually have greater understanding of horse management and breed their animals with clear economic objectives, resulting in an increase in selective intensity and the early replacement of mares by genetically superior ones. It has also been demonstrated that there is a conflict between the use of horses for sport and the development of gestation which contributes to an increase in the variability of reproductive parameters, especially in mares (Gómez et al., 2020).

Numerous studies have shown, in practically all species, that reproductive characteristics are among those most affected by inbreeding depression (Charlesworth and Willis, 2009). In comparison with other livestock species, horses have higher inbreeding coefficients, due mainly to the use of breeding schemes focused on morphological traits (Perdomo-González et al., 2020) or sport performance (Todd et al., 2018; Azcona et al., 2020), the lack of genetic control of mating (Gómez et al., 2009), the search of a certain type of animal, or the use of breeds with very small effective population sizes. The historical change of average inbreeding coefficients for F3 and F found here is similar to the latest studies carried out with the total PRE population (Perdomo-González et al., 2020; Poyato-Bonilla et al., 2020). The ongoing change of the average inbreeding values reflects the greater frequency of homozygous loci and loss of genetic variability. However, although both F and F3 have decreased in recent decades, F3 has shown a lower decrease than F. This implies that the reduction in F is probably due to the breeders’ concern, who avoided as far as possible the most inbred matings as opposed to previous decades when they sought to maximize the genetic similarity with emblematic ancestors. This also explains the fact that there is increasingly less difference between F and Fk. Therefore, Fk has values intermediate between F3 and F, which coincides with similar results obtained in Romanov sheep (Vostry et al., 2018), where Fk values were also intermediate. The degree of relatedness among the reproducers increased over recent generations, whereas the number of mares selected for reproduction decreased. This can be attributed to an increase in selection pressure in the last 20 yr and/or to an increase in the number of mares dedicated to equestrian sports instead to breeding.

Several studies examining the relationship between equine reproduction and inbreeding (Kutsal, 1960; Krashnikov et al., 1966; Kownacki and Jaszczak, 1968; Geurts, 1970) have shown the existence of inbreeding depression in traits related to equine fertility. However, Mahon and Cunningham (1982) found no significant relationship between the inbreeding coefficients of mares and their reproductive success in Thoroughbreds, although this may be due to their very low average inbreeding levels (*F* = 0.01). In contrast, Fomin et al. (1982) observed a decrease in conception and foaling rate with increased inbreeding coefficients in trotter mares. Similar results were reported in studies of Przewalski (Bouman, 1977) and Arab horses (Shemarykin and Vysokilova, 1986). Moreover, the percentage of unsuccessful matings was also affected by the inbreeding coefficient of potential offspring in Campolino horses (Fonesca et al., 1977), increasing from 25% for outbred matings to 60% from closely related matings. Recent studies have analyzed the kinship between the breeders (Sairanen et al., 2009), the effects of inbreeding on populations with extremely high inbreeding levels (Kjöllerström et al., 2015), other systematic effects on fertility (Müller-Unterberg et al., 2017; Ewert et al., 2018), and the genetics of reproductive performance (Gómez et al., 2020; Mantovani et al., 2020). To evaluate the effects of inbreeding on the studied traits, we calculated the regression coefficients between the inbreeding coefficients of the inbred mares (F3, Fk, and F) and their reproductive phenotypic values. Our results show the existence of significant inbreeding depression for all the reproductive traits. It is worth noting that the regression coefficients for Fk were as strong as those for *F* even though the Fk values were lower than those for *F* in the evaluated population. This shows that Fk can be considered a more sensitive parameter, in comparison to F3 and F, when evaluating inbreeding depression. However, the regression coefficients for F3 were less significant than for *F* or Fk and is therefore less useful for detecting inbreeding depression. This negative relationship between inbreeding coefficients and reproductive traits and efficiencies can be explained by the genetic load of deleterious alleles not being dissipated by natural or artificial selection in the breed or by the decreased heterozygosity for alleles at loci with a heterozygote advantage.

The estimates of heritability obtained in our study were similar to those reported by Gómez et al. (2020) for PRE mares, 0.15, 0.04, 0.14, 0.08, and 0.11 for AFF, I12, AIF, ALF, and PL, respectively. The estimates diverged most, albeit narrowly, in AFF. Our results show the same magnitude as the heritability estimates of other Spanish horse populations such as Arab (0.20) or Anglo Arab (0.16) (Gómez et al., 2020), but lower than those estimated for Spanish Sport Horse (0.32) (Gómez et al., 2020) or Thoroughbred (0.38) (Taveira and Dias, 2007). Heritability for FN (0.08) was similar to those previously reported in Warmblood horses (0.12) (Wolc et al., 2009) but higher than those in Standardbred (0.01) and Finn horses (0.03) (Sairanen et al., 2009). Nevertheless, changes in heritability estimates were expected due to the inclusion of the inbreeding load variance component to its calculation.

In this study, we have used a methodology that permits the joint estimation of the additive genetic components of a trait together with the inbreeding depression load ratios (Varona et al., 2019). Although heritability is related to the variability expressed in the population, inbreeding depression load ratios measure the potential variability, whether expressed in the population or not. In fact, the total value cannot be expressed, because if an animal is 100% inbred with an ancestor, it cannot be fully inbred with other ancestors. The inbreeding depression load ratios, obtained for an *F* value of 10%, ranged from 0.06 (PE6) to 0.17 (AFF), with similar magnitude that those studies that had analyzed inbreeding depression load ratios (Poyato-Bonilla et al., 2020; Martinez-Castillero et al., 2021).

Our results reveal that, in a theoretical population where all individuals had an inbreeding coefficient of 0.1 from one ancestor, the variability caused by the inbreeding depression load may surpass the variability caused by the additive genetic effects, which agree with those obtained by Martinez-Castillero et al. (2021) analyzing fertility traits in Brown Swiss cattle. Even that, to date, the studies on inbreeding depression load ratios (Casellas, 2018; Varona et al., 2019; Poyato-Bonilla et al., 2020; Martinez-Castillero et al., 2021) have reported low ratios for the inbreeding depression load, which, added to the wide variability of this parameter, establishes very wide confidence limits.

Inbreeding depression load in a population is dynamic over time. New deleterious recessive alleles arise continuously by mutation and these alleles are usually lost over time by natural and/or artificial selection and genetic drift (Hedrick and Garcia-Dorado, 2016). In addition, inbreeding depression is generally higher for traits associated with biological efficiency such as viability and reproductive ability, because there is more dominance at the loci affecting these traits than at the loci affecting others. The analysis of the change in IL over the years in the PRE evaluated population shows that the variation is not the same for all traits and that the effect of IL on the performance in inbred descendants is highly variable. According to our results, individuals with IL lower than −0.54, −0.47, and −0.36 for AFF, I12, and AIF, respectively, transmit a reduction in the phenotypic value of its inbred descendants for these traits, that is, they improve the mare’s reproductive aptitude. However, individuals with IL higher than 0.11, 3.40, 0.22, 0.78, 1.34, and 1.44 for FN, ALF, PL, GE, PE3, and PE6, respectively, will transmit an increase in the phenotypic value of its inbred descendants (mares with better reproductive aptitude). For instance, an individual with a Fij equal or higher than 10% from a single, specific, common ancestor with an IL of −0.54 will show precociousness at AFF in comparation to its contemporaneous individuals. Specifically, 1.61%, 19.58%, and 39.03% of the analyzed population had IL lower to the slope for AFF, I12, and AIF, respectively, and 29.09%, 8.12%, 1.52%, 12.09%, 2.04%, and 19.56% of the analyzed population had IL values above than the slope for FN, ALF, PL, GE, PE3, and PE6, respectively. Thus, common ancestors with a favorable IL could be favored in breeding programs and those with unfavurable values avoided. This introduces new possibilities for population breeding, selection indexes, and management strategies to the present strategy of avoiding or limiting inbreeding (Martinez-Castillero et al., 2021).

Related animals can be mated with the aim of increasing or decreasing a certain character, and the result also depends on the partial inbreeding and inbreeding depression load of the common ancestors. For this reason, correlations between IL and BV are of great interest. In our study, all the significant Pearson’s correlations between BV and IL for the same trait were positive or close to zero. In general, positive correlations indicate that horses with higher breeding values cause a particularly positive inbreeding effect in their inbred descendants, as in the case of FN, ALF, PL, GE, PE3, and PE6. However, for AFF, I12, and AIF, positive correlation means that horses with higher breeding values cause a negative inbreeding effect in their inbred descendants.

The Pearson’s correlation between BV and IL for different traits were mostly positive and of a low to medium magnitude, which suggests that, in general, individuals with high genetic values for reproductive traits will not cause much inbreeding depression. However, the strongest correlations, mainly between IL_AFF, IL_ALF, and IL_PL and the other traits, indicate that it is crucial to take into account the fact that the selection of horses based on their additive genetic values for one reproductive trait could lead to different inbreeding depression loads for the other reproductive traits.

The correlations between IL for different traits showed positive values and were of a moderate to high magnitude. Positive correlations indicate that horses with a high positive inbreeding depression load for a given trait would also have a positive inbreeding effect on their inbred descendants for the other trait. In contrast, a negative correlation indicates that horses with a high, positive inbreeding depression load for given reproductive traits would have a negative inbreeding effect on their inbred descendants for the other trait. According to our results, exerting selection pressure on horses with high, positive inbreeding depression load values for IL_AFF, IL_AIF, IL_FN, IL_GE, IL_PE3, and IL_PE6 will have, in general, a positive effect for most traits, although exerting selection pressure on horses with high, positive inbreeding depression load values for IL_ALF and IL_PL will have, in general, a negative effect on the rest of the traits.

In conclusion, our results demonstrate the importance of taking the potential inbreeding depression load into account for reproductive traits in order to improve reproductive fitness in the PRE breed, and that the result of mating related animals to increase or decrease traits not only depends on their additive genetic value, but also on their partial inbreeding and the inbreeding depression load genetic values of the common ancestors. In fact, our results indicate that the inbreeding depression load values have a greater influence than additive genetic values on the fertility traits of inbred animals, when analyzing animals with a large Fij from a specific ancestor. For this reason, understanding the individual inbreeding depression load genetic values of stallions and mares for a particular trait is of great interest to the PRE Breeding Programme, where the detrimental or beneficial repercussions of alleles from certain lineages would allow breeders to make a better selection of breeding animals.

## Abbreviations

BV: breeding value
F: Wright’s coefficient of inbreeding
Fij: partial inbreeding coefficient
Fk: Kalinowski ancestral inbreeding coefficient
F3: recent inbreeding coefficient at 3rd generation
GE: general efficiency
IL: inbreeding depression load genetic value
PL: productive life
